# Enhancing Reciprocating Wear Resistance of Co37Cr28Ni31Al2Ti2 Spark Plasma Sintered Medium-Entropy Alloy via TiC Addition

**DOI:** 10.3390/ma18020442

**Published:** 2025-01-18

**Authors:** Yubo Zhao, Wenbo Ma, Oleksandr Tisov

**Affiliations:** School of Aerospace Engineering, Xi’an Jiaotong University, West Xianning Road 28, Xi’an 710049, China; linhaideshu@stu.xjtu.edu.cn (Y.Z.); mawenbo202207@163.com (W.M.)

**Keywords:** medium-entropy alloy, tribological properties, wear resistance, CoCrNi-TiC alloy, abrasive wear

## Abstract

The aim of this paper is to investigate the effect of TiC addition on the microstructure, microhardness, and wear resistance of the medium-entropy alloy Co37Cr28Ni31Al2Ti2, which is suitable for applications in aerospace, automotive, and energy industries due to its high strength and wear resistance. The samples containing 0, 10, 20, and 40 wt.% of TiC were synthesized. The alloy’s microstructure changes significantly with the addition of TiC particles: they are uniformly dispersed in the FCC matrix, effectively increasing the Vickers hardness from 439 HV for the base alloy to 615 HV for the 40% TiC alloy. The four alloys were subjected to reciprocating dry sliding friction tests at loads of 2 N, 5 N, and 10 N. The wear volumes of the base alloy at these loads were 2.7 × 10^7^, 4.6 × 10^7^, and 1.1 × 10^8^ μm^3^, respectively. The experimental results indicate that adding TiC greatly improves the wear resistance of the alloy by increasing the hardness and forming an oxide protective film. This study highlights the potential for developing alloys with excellent tribological properties for demanding application scenarios.

## 1. Introduction

Modern engineering industries constantly require new materials with more advanced properties than years before. “Classical” materials have almost exhausted their properties, and the prospects for their improvement are not so much. This is why, in recent decades, new groups of materials have emerged, such as oxide dispersion-strengthened alloys, nano-modified composite materials, medium- and high-entropy alloys. Medium-entropy alloys (MEAs) have attracted much attention due to their excellent high-temperature mechanical properties and oxidation resistance. At the same time, their composition is quite simple; they mainly contain three or four components.

In 2004, Yeh et al. [1] and Cantor et al. [2] proposed a new concept of alloy design by mixing multiple elements in equimolar or near-equimolar fractions [3], which stimulated the exploration of broader compositional space. Due to the unique four core effects of high entropy, slow diffusion [4], severe lattice distortion, and the cocktail effect [5,6,7,8], MEAs have excellent comprehensive properties, such as toughness, high-temperature resistance, oxidation resistance, and abrasion resistance [9,10,11,12], and they have important potential applications as high-performance friction materials [1,4,5,6,7,8,9,10,11,12,13].

Several studies have shown that introducing ceramic particles into the single-phase matrix of MEAs is an effective way to improve the mechanical properties [14,15,16,17,18,19,20]. For example, by adding 7% TiC (10 μm) and 3% SiC (10 nm) ceramic particles in CoCrFeMnNi high-entropy alloy (HEA), the compressive yield strength of the alloy was increased to 1480 and 1600 MPa, respectively [21]. Adding a certain amount of carbon to the MEA [22] or HEA [9] effectively improves the material’s wear resistance. In addition, by adding ceramic particle Al_2_O_3_ to the base alloy [23], the crystal lattice is refined, and the mechanical properties of the alloy are successfully improved. From this evidence, the hard refractory particles are used to increase the tribological performance of the materials. TiC is often used as a strengthening phase because of its high hardness (31 GPa), high melting point (3140 °C), good wettability, and chemical stability [24,25,26,27,28,29,30].

Wang et al. [31] used ball milling combined with the spark plasma sintering (SPS) method to prepare Fe40Mn40Cr10Co10 MEA modified by a TiC volume fraction of 10%. The results showed that the compression strength of the alloy was increased from 1571 to 2174 MPa, and the hardness was increased from HV 320 to HV 872. By adding different contents of TiC to the CoCrNi MEA, the hardness, corrosion resistance, and other properties of the base alloy were improved [32]. In addition, Zhang et al. [33] successfully improved the corrosion resistance of the primary alloy by introducing TiC into Al0.1CrNbSi0.1TaTiV refractory HEA. So, introducing TiC particles could effectively improve the mechanical properties of the medium-entropy alloy. It has been shown that the ex situ addition of ceramic phase can effectively improve the wear resistance of medium-entropy alloys [34,35,36,37], which provides a new idea for the research of wear-resistant materials. So far, the research on carbide modification of HEAs and simple MEAs has been sufficient. However, there are few studies on complex intermediate-entropy alloys. Studies on the carbide modification, friction, and wear testing of potential MEAs are even fewer. Jiang et al. [38] designed non-equiatomic single-phase FCC Ni2Co1Fe1V0.5Mo0.2 MEA. Although its unique multi-principal component structure breaks the strength–plasticity paradox, it exhibits obvious strain hardening and excellent plasticity in the temperature range of 25–800 °C. Jiang et al. [21] also designed non-equiatomic alloy Co37Cr28Ni31Al2Ti2 MEA. A mechanical properties test has proved that this MEA has good wear resistance. However, the modification treatment of this alloy is less, and the material’s wear resistance can only be improved by adding a high amount of carbon. In addition, there are few friction experiments, and it is difficult to summarize the properties of such non-equiatomic MEAs systematically. Therefore, this study attempts to introduce TiC via ex situ addition and to explore the effect of different contents of TiC on the Co37Cr28Ni31Al2Ti2 MEA.

Spark plasma sintering (SPS) is a promising technology for manufacturing functional ceramic materials and alloys due to its ability to preserve the fine microstructure and achieve rapid sintering at low temperatures. Shichalin et al. [39] synthesized a mesoporous adsorbent based on calcium silicate (CaSiO_3_) using SPS at 1000 °C, which was used to immobilize cobalt 60Co radionuclides in durable CaCoSi_2_O_6_ ceramic matrices. The resulting material exhibited high compressive strength (481 MPa) and microhardness (~9.81 GPa), demonstrating the potential of SPS for producing high-performance materials. Therefore, SPS technology is also employed in this study to prepare a series of MEAs.

The aim of this study is to systematically investigate the effect of TiC contents, ranging from 10–40 wt.%, on the tribological properties of the Co37Cr28Ni31Al2Ti2 medium-entropy alloy. Adjusting the TiC content alters the alloy’s microstructure and hardness, which, in turn, significantly impacts its wear resistance. This research provides a novel contribution by focusing on the synergistic effects of TiC dispersion and the formation of protective oxide films in enhancing wear performance. These findings offer deeper insights into tailoring the tribological properties of medium-entropy alloys for demanding industrial applications, advancing the understanding of TiC-reinforced alloy systems.

## 2. Materials and Methods

### 2.1. Materials Preparation

The Co37Cr28Ni31Al2Ti2/(TiC)x (x = 0, 0.1, 0.2, 0.4) alloy samples were prepared by adding varying weight percentages of TiC to the MEA, with the specific nominal compositions listed in Table 1. The 15–50 μm MEA powders (alloy 1 in Table 1), serving as the solid solution matrix, were purchased from Gaoxin (Beijing, China) and have a spherical morphology. The 15–50 μm TiC particles, acting as the strengthening phase, were supplied by Zhongyue Xindun (Shijiazhuang, China) and also exhibit a spherical morphology.

The powders were equilibrated using an electronic balance FA2204N (JINGHAI, Shanghai, China) with an accuracy of 0.1 mg and then mixed in a planetary ball mill QM-3SO4 (CHISHUN TECH, Nanjing, China) placed in a vacuum tank filled with argon at normal pressure. Mixing was carried out using 6 mm steel balls with a powder-to-ball ratio of 1:10, and the rotational speed was set at 300 revolutions per minute for 360 min.

The formulated powders were subjected to spark plasma sintering (SPS) using HP D 10 SI (FCT, Frankenblick, Germany). When the vacuum reached 5 × 10^−3^ Pa, argon was injected into the furnace, which was then heated and pressurized at 75 °C/min and 2.1 MPa/min until the temperature and pressure met 1100 °C and 30 MPa, respectively, and finally held in this state for 8 min.

Once the alloys were sintering completed, the resulting 30 × 4 mm discs were cut into 15 × 8 × 4 mm blocks for subsequent testing using the medium-speed wire cut electrical discharge machine HA400U (Sankuang, Suzhou, China). In addition, samples were mechanically ground with 2000 grit SiC sandpaper and polished with 3 μm and 1 μm diamond suspensions using a VP 430 automatic polishing machine (Trojan Material Technology Co., Ltd., Suzhou, China) before testing. The surface roughness of the test samples was Ra = 0.07 ± 0.02 μm, Rz = 0.8 ± 0.01 μm.

### 2.2. Microstructure Characterization

The phase composition of the sintered samples was analyzed by an X-ray diffractometer D8 Advance (Bruker, Karlsruhe, Germany) with a scanning range of 2θ = 20…100° at the speed of 5°/min. The microstructure and elemental composition of the alloys were observed using the scanning electron microscope (SEM) EVO10 (ZEISS, Jena, Germany) equipped with an Oxford energy dispersive spectrometer (EDS) analyzer (Oxford Instruments, Abingdon, UK). In addition, the surface porosity of different alloys can be calculated using ImagePro Plus 6.0 software (Media-Cybernetics, inc., Rockville, MD, USA).

The alloy’s porosity was determined using the hydrostatic balance method. The samples’ dry weight (in grams), floating weight (in grams), and wet weight (in grams) were also determined with an accuracy of 0.1 mg. To ensure full saturation of the sample pores and obtain accurate wet weight measurements, the samples were immersed in water at 100 °C for 2 h before testing.

### 2.3. Mechanical Properties

Hardness tests were carried out on the HVST-1000Z (JUNDA, Guangzhou, China) Vickers hardness tester with a load of 2 N and a holding time of 10 s. The average of five measurements was taken to characterize the microhardness of alloys.

The wear behavior of alloys under ambient atmospheric conditions was simulated using the RTEC MFT5000 multifunctional tribometer (RTEC instruments, Silicon Valley, CA, USA), which has a ball-on-plate reciprocating configuration. The void-free ZrO_2_ balls with a diameter of 6 mm were used as the friction partner. In addition, the loads used for friction simulation are usually chosen according to the hardness of the tested material. Specifically, when the hardness is low, a load of 2 or 5 N is usually applied, whereas a load of 10 or 20 N is preferred for harder materials. Referring to the literature [40], the values of normal force used in this work were 2 N, 5 N, and 10 N. The friction time was 30 min, with a reciprocating stroke of 5 mm and a frequency of 5 Hz. Upon completion of friction tests, the alloys’ volume loss and wear scar profiles were studied to characterize their wear resistance using the RTEC UP3000 optical profilometer (RTEC instruments, Silicon Valley, CA, USA).

## 3. Results and Discussion

### 3.1. XRD Analysis

By comparing the X-ray diffraction (XRD) data of the four alloy samples with standard diffraction card patterns, it was observed that the XRD pattern of alloy 1 corresponds to the standard card 15-806, which represents the FCC structure of cobalt (Co) from the International Crystal Diffraction Database. In the case of alloy 2, 3, and 4, the XRD patterns not only display the presence of the FCC phase but also reveal diffraction peaks corresponding to the TiC phase. The residual peaks of these alloys match with card number 32-1383, which represents the FCC structure of TiC crystals, as listed in the International Crystal Diffraction Database.

**Figure 1:**
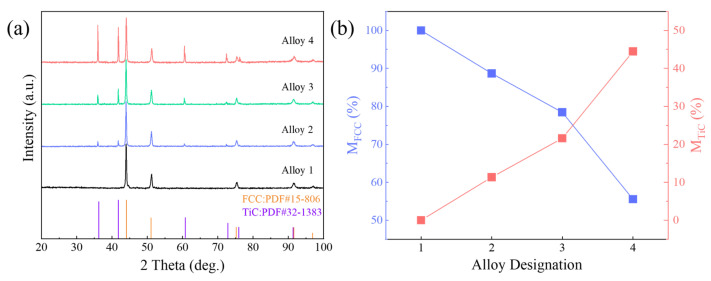
(**a**) XRD patterns and (**b**) content of TiC and FCC phases of MEA/(TiC)x (x = 0, 0.1, 0.2, 0.4).

The results of XRD analysis of Co37Cr28Ni31Al2Ti2/(TiC)x (x = 0, 0.1, 0.2, and 0.4) are shown in Figure 1. The results indicate the formation of a single FCC phase in alloy 1 in Table 1, which agrees with the results published in [32]. Upon the incorporation of varying concentrations of TiC, obvious TiC peaks appeared in the diffraction patterns of alloy 2–4, indicating the structures change from a single-phase FCC solid solution to bimodal FCC + TiC. Since no chromium carbide was detected, it proves that the TiC powders used were not contaminated with Carbon.

### 3.2. Microstructure

**Figure 2:**
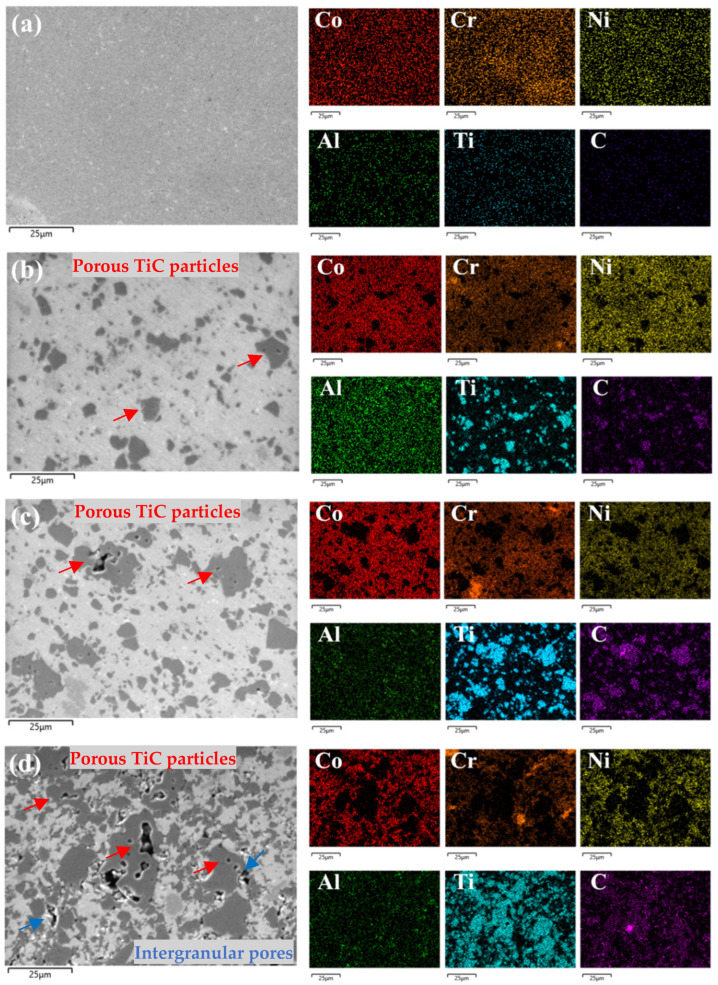
SEM and EDS images of MEA/(TiC)x (x = 0, 0.1, 0.2, 0.4): (**a**) alloy 1; (**b**) alloy 2; (**c**) alloy 3; (**d**) alloy 4. Red arrows show pores inside the TiC grains; blue arrows show pores between HEA particles and TiC grains.

Figure 2 illustrates the SEM and EDS results of Co37Cr28Ni31Al2Ti2/(TiC)x (x = 0, 0.1, 0.2, and 0.4), where the TiC particles are uniformly distributed in the MEA matrix without any dissolution or precipitation, indicating that the alloys are fully sintered. As the carbide content increases, it is clear that more TiC particles are located between the initial MEA particles. Moreover, the porosity of the samples also increases with increasing carbide, which is mainly due to the intrinsic cavities in the TiC particles (Figure 2b–d, red arrows), whereas the gap between the TiC and the initial MEA is not significant (Figure 2d, blue arrows).

The actual element composition of the MEAs was calculated according to the EDS results (Table 2). The error in the C content determination is because we used conductive carbon adhesive to fix the samples inside the microscope chamber. Figure 2 gives clear evidence that the powders are evenly mixed; solid solution elements do not demonstrate clustering, and the distribution of elements forming TiC correspond to carbide positions.

The dry weight, floating weight, and wet weight of the four alloys were measured using the hydrostatic balance method (Table 3). Based on these measurements, the porosity of each alloy was calculated.

The SEM images of the alloys were processed and analyzed using image analysis software (ImagePro Plus 6.0) to obtain the proportion, average size, and maximum particle size of TiC aggregates in the different alloys, as summarized in Table 4. As the TiC content increases, TiC aggregates gradually form in the MEAs. The proportion of these aggregates grows, and the size of the largest aggregate also expands. Moreover, the average grain size shows an increase with higher carbide content, and the alloy’s porosity also rises as the carbide concentration increases.

**Figure 3:**
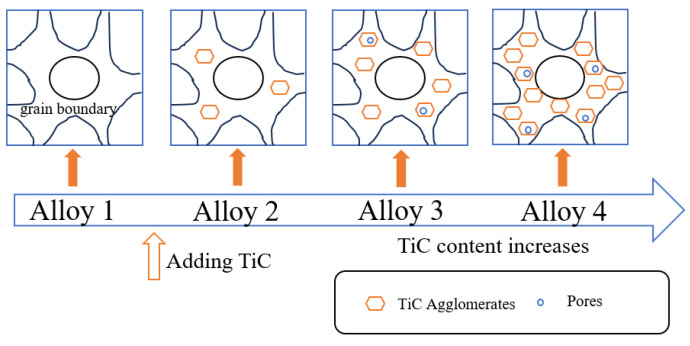
Diagram of microstructure evolution with increasing TiC content in MEA.

Figure 3 summarizes the distribution and accumulation process of TiC in the MEAs based on the previous analysis and verification. At low TiC content, TiC particles are well-dispersed in the alloy matrix, with no noticeable agglomeration (Figure 3, alloy 2). As TiC content increases, excess TiC particles aggregate into clusters, leading to a two-phase structure with the FCC matrix and TiC (Figure 3, alloy 3). With further increases in TiC content, the size of the largest TiC aggregates grows, and the voids between particles also expand, resulting in a more distinct two-phase structure (Figure 3, alloy 4).

### 3.3. Hardness and Wear Resistance

**Figure 4:**
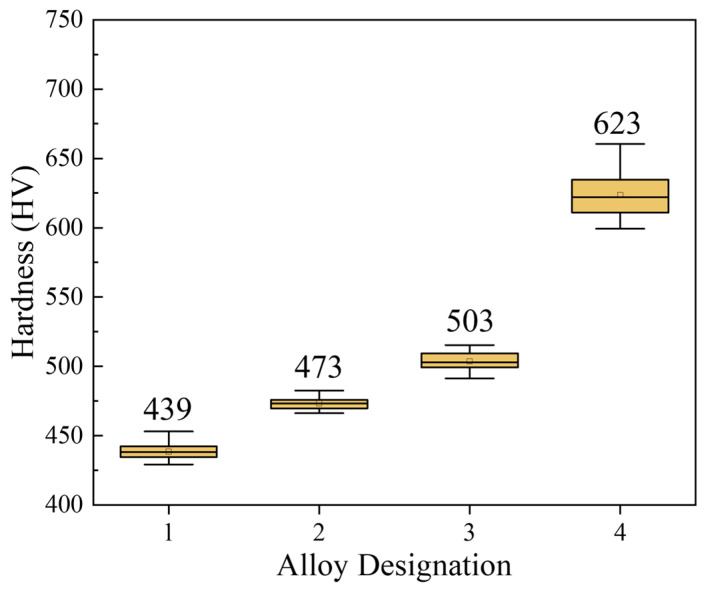
Variation of Vickers hardness of MEA/(TiC)x (x = 0, 0.1, 0.2, 0.4).

The Vickers hardness of the alloys was measured at room temperature. The microhardness increased with the addition of TiC, as shown in Figure 4. TiC increased the microhardness of alloys 2, 3, and 4 to HV 473, HV 503, and HV 623, respectively. The hardness of alloy 4 increased by 41.9% compared to alloy 1 (HV 439). The addition of TiC in the alloy also increased the Vickers hardness of the alloy. Comparing the hardness tests already reported for MEAs [32], the hardness of the MEA with added TiC is significantly higher than that of the conventional MEA (CoCrNi) with the same content of TiC. Moreover, Rabinowicz’s abrasive wear model [41] demonstrates that an increase in hardness is associated with a reduction in wear rate (WR). Gahr [42] found that the common steel and cast-iron materials containing hard carbides had improved wear resistance. In the conventional Fe-Cr-C wear-resistant alloy system, the increase in hardness and wear resistance can be achieved by adjusting the content, distribution, and size of M_7_C_3_ carbides [43,44,45,46]. Therefore, higher hardness is an important factor in improving wear resistance.

**Figure 5:**
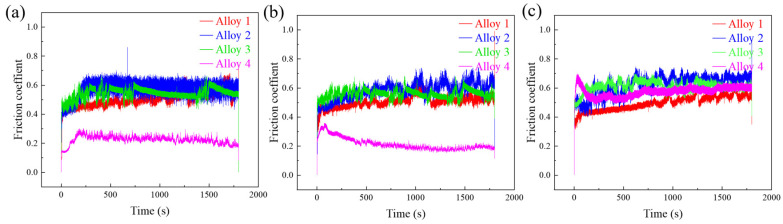
Typical CoF curves at loads of (**a**) 2 N, (**b**) 5 N, and (**c**) 10 N.

Typical coefficient of friction (CoF) curves of four alloys tested at room temperature under contact force of 2, 5, and 10 N are given in Figure 5, and the average friction coefficients are shown in Table 5. The CoF curves of alloy 1 at 2 N load and alloy 2 at 2 N and 5 N loads showed typical periodic fluctuations. The CoF curves of these two samples at 10 N load show a clear jagged behavior. Periodic localized fracture of the surface layer and periodic accumulation and elimination of wear debris are believed to be responsible for this difference [47,48].

In contrast, alloy 4 exhibited a significant increase in CoF under 10 N load, rising to 0.5730, more than twice the values at 2 N (0.2251) and 5 N (0.2137). This sharp increase can be attributed to a combination of factors. First, the higher TiC content in alloy 4 increases the material’s brittleness, making it more susceptible to microcracking and surface damage under higher loads. Second, the elevated load intensifies the wear process, causing more significant disruption of the oxide protective film, which is crucial in reducing friction. Additionally, the higher load leads to increased accumulation of abrasive wear debris, which further exacerbates frictional forces and contributes to the sharp rise in CoF.

**Figure 6:**
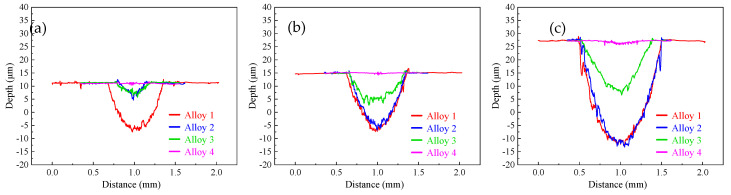
Cross−sectional two−dimensional profiles at loads of (**a**) 2 N, (**b**) 5 N, and (**c**) 10 N.

Based on the CoF curves, it can be observed that the coefficients of friction of alloy 4 are more stable. Compared with alloy 1, the wear losses (Figure 6a–c) of the remaining three alloys with TiC addition are reduced to some extent at different loads. And there is a significant reduction in the wear loss of the samples as the content of TiC increases, which should be attributed to the high hardness and wear resistance of TiC itself and the presence of oxides produced during the friction process. In the friction wear tests, the oxides are more difficult to be worn, resulting in a reduction of the actual contact area [49].

According to the 2D topography, the specific depth (Table 6) and width (Table 7) of the wear marks of the four materials under different loads were calculated. The width and depth of the wear track gradually increase with the increase in the load. The addition of TiC effectively reduces the depth and width of the wear mark, which is particularly obvious on alloy 4.

**Figure 7:**
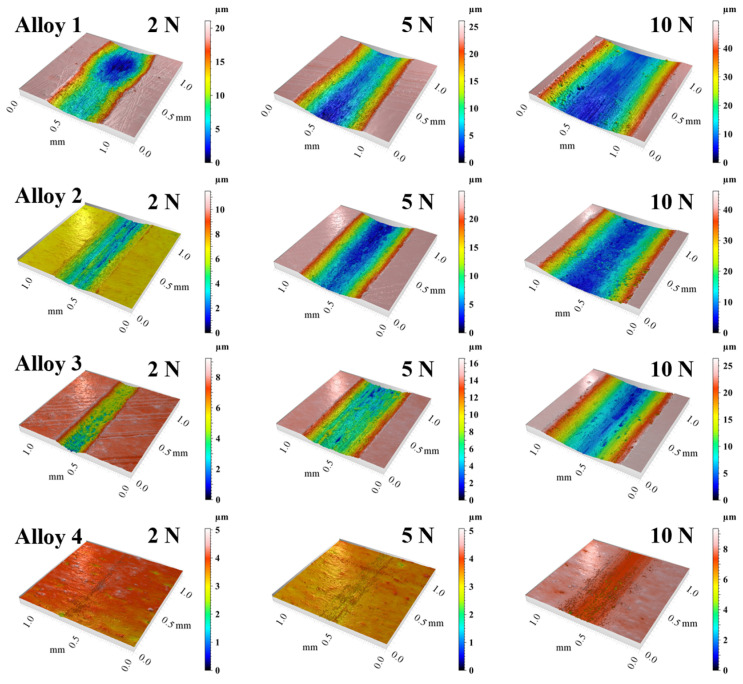
Three-dimensional topographies of wear tracks for MEA/(TiC)x (x = 0, 0.1, 0.2, 0.4).

Figure 7 shows the 3D morphology of the wear track. Under the same load, the wear scars of the MEA sample are smoother and wider, while the scars of the three samples with TiC addition are rougher and narrower. The 2D and 3D morphology of the wear marks of the samples showed widening and deepening of the wear trajectories.

**Figure 8:**
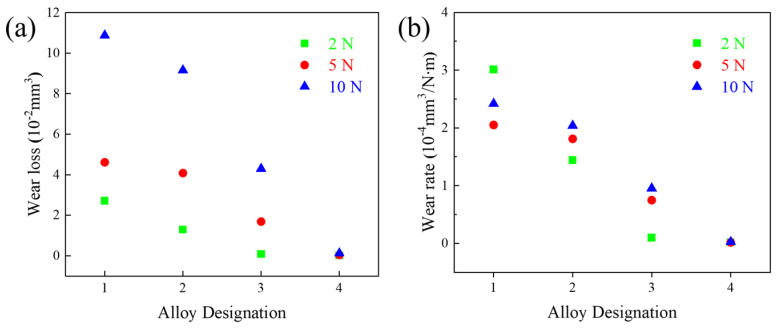
(**a**) Volume loss and (**b**) wear rates of the MEA/(TiC)x (x = 0, 0.1, 0.2, 0.4).

In addition, Figure 8a shows the volume loss of the alloys. The average volume loss of alloy 1 was the highest for all three loads: 2.712 × 10^−2^ ± 1.62 × 10^−3^ mm^3^, 4.613 × 10^−2^ ± 2.75 × 10^−3^ mm^3^, and 1.088 × 10^−1^ ± 5.89 × 10^−3^ mm^3^, respectively. The volume loss of all four alloys continued to increase as the applied load increased to 10 N. For all samples, the volume loss increased with the load, and at the same time, the wear volume of the samples decreased significantly with increasing TiC content. The volume loss of alloy 4 is much lower (1.208 × 10^−3^ ± 8.21 × 10^−5^ mm^3^) compared to that of alloy 1 (1.088 × 10^−1^ ± 5.89 × 10^−3^ mm^3^).

Figure 8b shows the wear rates of the alloys. The wear resistance of the alloys increased significantly with increasing TiC content. The WR of alloy 1 at different loads (2 N, 5 N, and 10 N) were 3.01 × 10^−4^ mm^3^/N·m, 2.05 × 10^−4^ mm^3^/N·m, and 2.42 × 10^−4^ mm^3^/N·m, respectively, which are comparable to the reported MEAs [9,37,50,51,52,53]. On the other hand, alloy 4 exhibits a very low WR of 2.0 × 10^−6^ mm^3^/N·m, 1.73 × 10^−6^ mm^3^/N·m, and 2.689 × 10^−6^ mm^3^/N·m, respectively, for different loads. Thus, TiC addition provides better wear resistance than cast MEAs of similar compositions and is superior to the reported MEAs after surface treatment [54,55,56,57].

Alloy 4 exhibited the lowest wear at all loads, demonstrating that adding TiC significantly enhanced the wear resistance of the materials. At low loads, the wear volumes of all materials are relatively low, and all the alloys modified with TiC are greatly superior to alloy 1. This is because, at low loads, the wear mechanism is primarily characterized by slight adhesive and abrasive wear. The presence of TiC increases the material’s hardness and promotes the formation of a protective oxide layer during the friction process. This oxide layer is more difficult to wear away, reducing the contact area between the friction surfaces, thereby improving wear resistance.

**Figure 9:**
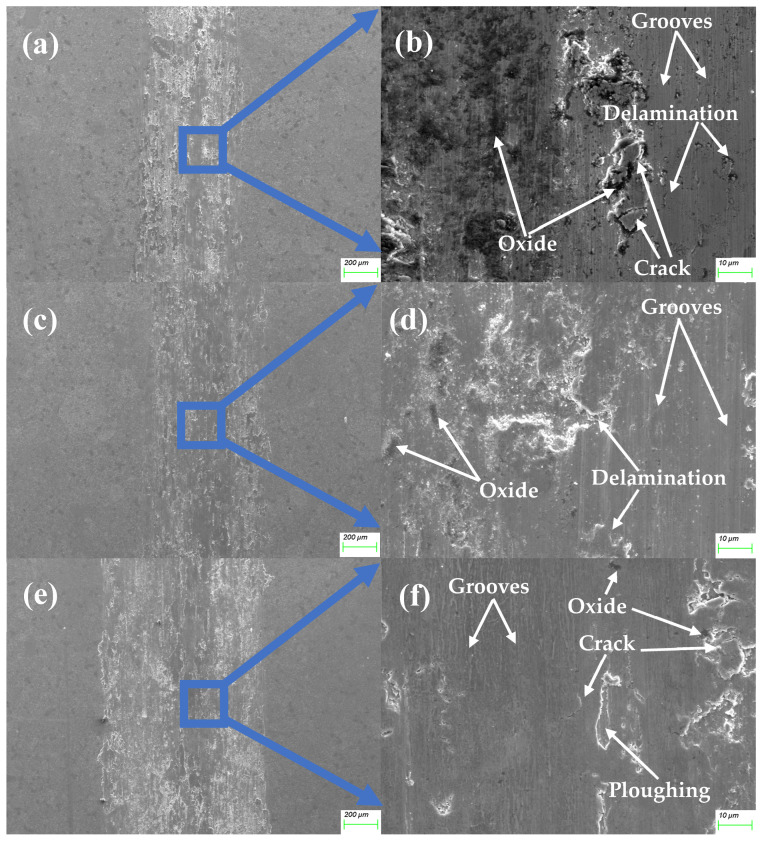
The morphology of the friction surface of the MEA/(TiC)x (x = 0) tested at 2 N (**a**,**b**), 5 N (**c**,**d**), and 10 N (**e**,**f**).

Figure 9 shows the wear track morphology of alloy 1. This material exhibits the highest wear loss across all loads, characterized by numerous grooves, cracks, and delaminations on the surface. The dark areas observed in Figure 9b indicate friction-induced oxidation, as the resulting oxides, which are harder and more brittle than the base material, contribute to the spallation seen in Figure 9d. This oxide spallation promotes significant abrasive wear, resulting in high surface roughness and pronounced wear scars that widen with increasing load, as shown in Figure 9e. The generation of frictional heat at higher loads facilitates surface oxidation, leading to oxide layer delamination and rapid peaks in the CoF graphs. Additionally, this heat softens the material, causing extensive plastic deformation, as evidenced in Figure 9f, thereby exacerbating wear and oxidation processes. The primary wear mechanisms are severe abrasive and oxidative wear, with clear evidence of grooves and oxides on the material surface.

**Figure 10:**
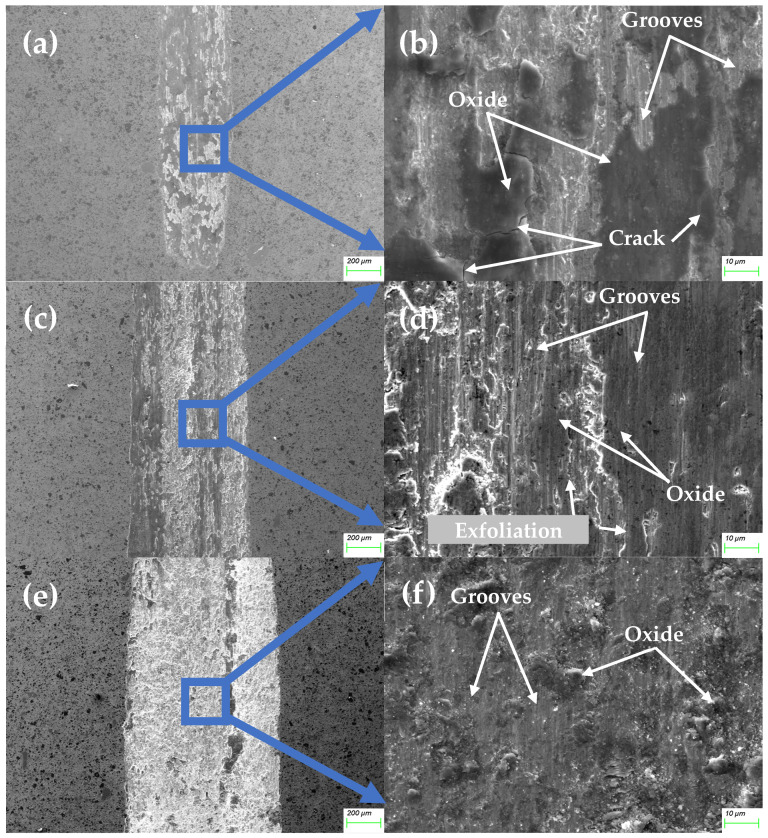
The morphology of the friction surface of the MEA/(TiC)x (x = 0.1) tested at 2 N (**a**,**b**), 5 N (**c**,**d**) and 10 N (**e**,**f**).

Alloy 2 exhibits significantly reduced wear at a 2 N load, but the wear remains substantial at higher loads (5 N and 10 N). Compared to the unmodified material, the friction surface at all loads shows a greater coverage of oxides, particularly at 2 N (Figure 10b). However, the protective effect of the oxides diminishes at higher loads, leading to an increased WR comparable to that of the unmodified material. Figure 10c–f reveal more intensive abrasion than in the unmodified material, primarily due to TiC particles rather than fragments of the lost oxide layer. Notably, at 5 N, abrasion is particularly severe (Figure 10d), while at 10 N, the surface becomes more flattened due to higher contact pressure (Figure 10f), with some oxide debris embedded in the surface.

No plastic deformation was observed, indicating effective strengthening by TiC. However, with only 10% TiC content, particles separate from the binder during friction, contributing to abrasion and irregularities in the CoF curves. The primary wear mechanism at this composition is abrasive wear, evidenced by numerous furrows (Figure 10d), as the low TiC content limits the volume-strengthening effect. During friction, TiC particles detach from the surface and cause scratches, especially at higher loads, while the exposed metal surface undergoes oxidation, increasing oxidative wear. Nonetheless, adding TiC provides improved wear resistance compared to the base material, though the effect is limited.

**Figure 11:**
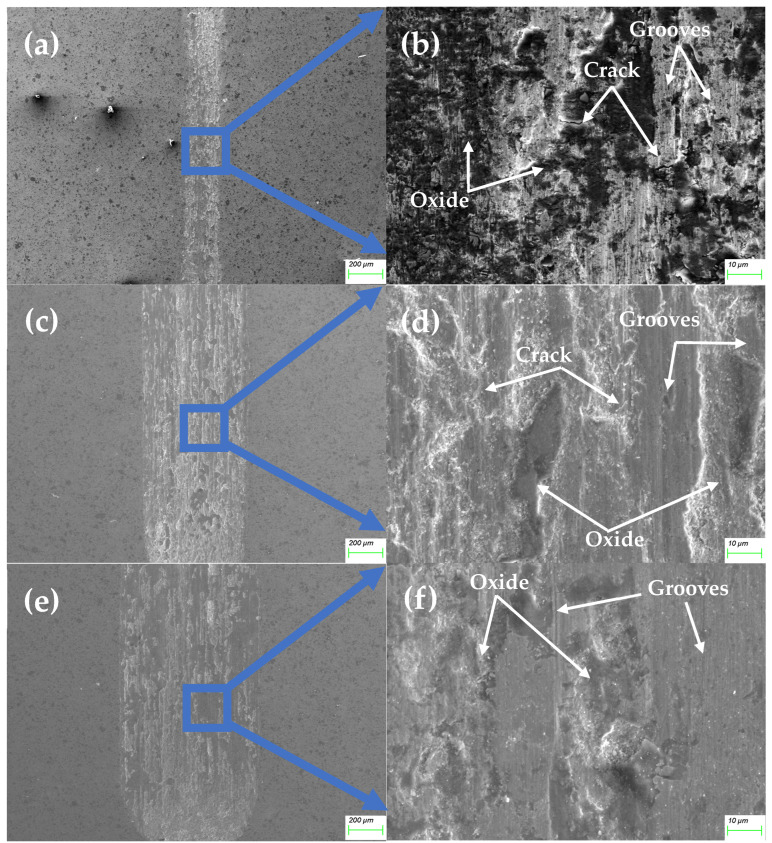
The morphology of the friction surface of the MEA/(TiC)x (x = 0.2) tested at 2 N (**a**,**b**), 5 N (**c**,**d**), and 10 N (**e**,**f**).

Figure 11 shows that alloy 3 significantly reduced wear at all loads, particularly at higher loads (5 N and 10 N), demonstrating good anti-wear performance. This intermediate behavior between the samples with lower and higher TiC content highlights the crucial role of TiC particles in enhancing wear resistance. Compared to Figure 10, the surface shows better coverage with an oxide film and reduced abrasive wear. TiC is more effective at this concentration, and the friction-induced oxidation layers are more stable, reducing wear.

However, like it was for alloy 2, TiC particles are not visible on the friction surface, possibly due to their spallation or coverage by wear debris. The compacted debris separates the friction surfaces, reducing adhesion and wear intensity. The primary wear mechanisms are abrasive wear combined with oxidative wear. The wear volume data indicate that adding 20% TiC significantly enhances wear resistance.

**Figure 12:**
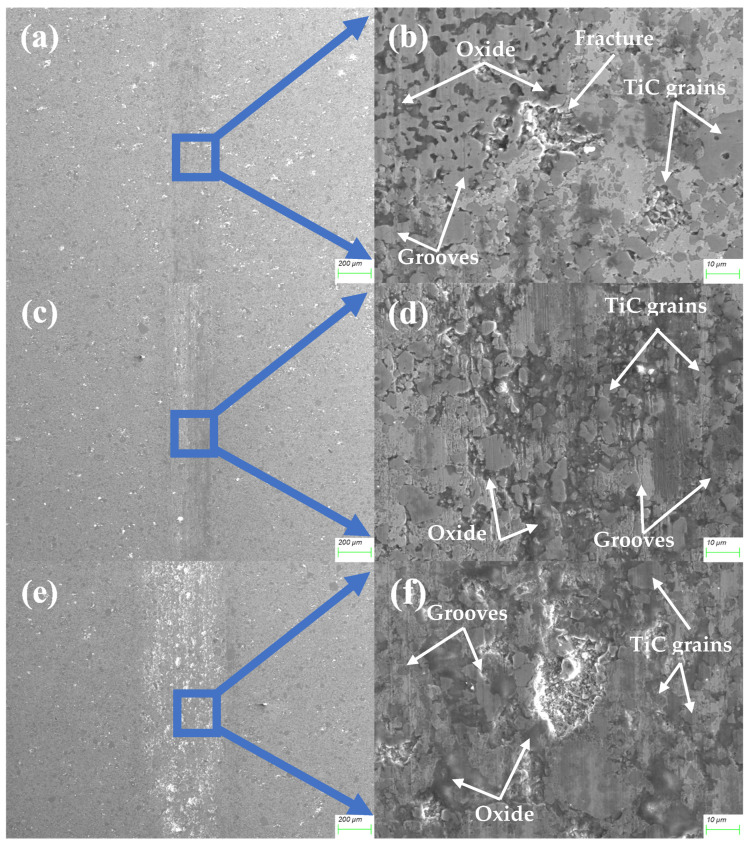
The morphology of the friction surface of the MEA/(TiC)x (x = 0.4) tested at 2 N (**a**,**b**), 5 N (**c**,**d**), and 10 N (**e**,**f**).

Alloy 4 exhibits the lowest wear at all loads, as shown in Figure 12. The friction surface is smooth, with no significant damage. The high TiC content changes the structure of the friction surfaces, and TiC particles are visible at all loads according to SEM images. (Figure 12b,d,f). The surface is partially covered with thin oxide layers between these grains, lacking grooves or scratches, although many TiC grains are scratched (Figure 12b,d,f).

TiC grains constitute a substantial portion of the friction surface. The metal phase shows minimal abrasion and no adhesion. The primary wear occurs between the zirconia ball and TiC particles, preventing the metal binder from significant friction. The metal phase between TiC grains exhibits some oxidation (Figure 12d,f), with oxidation spots overlapping scratches, suggesting that friction initiates with abrasion, followed by the oxidation of plastically deformed material. These oxide layers, coupled with TiC grains, reduce adhesion and the friction coefficient, resulting in a smooth and stable wear process.

The high TiC content significantly enhances the hardness and wear resistance, though it also leads to some porosity within the material, causing deeper holes on the friction surface at 10 N (Figure 12f). However, this has a negligible impact on surface wear, as evidenced by the lowest CoF, the smoothest track profile, and the lowest wear among all studied materials. The primary wear mechanism is slight abrasive wear with low-intensity oxidation wear.

In summary, the hardness and wear resistance of the MEAs increased significantly with increasing TiC content. Under low magnification, all TiC-added alloys demonstrated more uniform and clear wear trajectories, reducing severe material spalling and deep grooving phenomena. Wear mechanisms differed at different loads, with the alloys without TiC predominantly exhibiting adhesive, abrasive, and oxidative wear, whereas the TiC-added alloys showed predominantly abrasive wear and a certain amount of oxidative wear. The formed oxidation-type secondary surface structures supported with carbides embedded in ductile metallic matrices provide high surface strength and wear resistance [58,59,60,61]. The alloys with 20% and 40% TiC showed significant wear resistance at higher loads (5 N and 10 N), indicating that appropriate TiC content can effectively improve the materials’ wear and oxidation wear resistance.

## 4. Conclusions

- The medium-entropy alloy samples were well-sintered with a uniform structure. In the matrix of the alloy containing TiC, agglomerated carbides appear. As the TiC content increases, the carbide in the alloy changes from uniform particle distribution with no porosity to later carbide agglomeration with increased porosity. The major part of porosity is formed by internal voids inside TiC grains, and a minor part of it is located on the TiC-MEA interphase. The performed research proves the strong positive effect of TiC addition on the wear resistance of Co37Cr28Ni31Al2Ti2 MEA.
- The hardness of the basic alloy is 439 HV, which increases to 473 HV with 10% TiC, 503 HV with 20% TiC, and 623 HV with 40% TiC. The addition of TiC particles significantly enhances the hardness of the alloy.
- In tribological testing, the WR decreases significantly with increasing TiC content, indicating an improvement in wear resistance. The wear volumes of alloy 1 under loads of 2 N, 5 N, and 10 N are 2.712 × 10^−2^, 4.613 × 10^−2^, and 1.088 × 10^−1^ mm^3^, respectively. For alloy 2, the wear volumes under the same loads are 1.296 × 10^−2^, 4.080 × 10^−2^, and 9.164 × 10^−2^ mm^3^. The wear volumes for alloy 3 are 8.83 × 10^−4^, 1.686 × 10^−2^, and 4.298 × 10^−2^ mm^3^. The wear volumes for alloy 4 are 1.771 × 10^−4^, 3.93 × 10^−4^, and 1.208 × 10^−3^ mm^3^, respectively.
- The primary wear mechanisms for the basic alloy are adhesive wear and oxidative wear. Due to the lower hardness, significant plastic deformation and microcracks occur during friction. As the TiC content increases, the wear mechanism gradually changes to abrasive wear. A strong protective layer forms, particularly for high-TiC-content alloys, effectively reducing wear. The alloy with the highest TiC content (40% TiC) exhibits mild abrasive wear, significantly reducing wear volume and wear rate.

## Figures and Tables

**Table 1:** Nominal composition of Co37Cr28Ni31Al2Ti2/(TiC)x medium-entropy alloy: x = 0, 0.1, 0.2, 0.4.

Alloy No.	Elemental Composition, wt.%					
	Co	Cr	Ni	Al	Ti	TiC
1	38.87	25.98	32.48	0.96	1.71	0.00
2	34.99	23.38	29.23	0.86	1.54	10.00
3	31.10	20.79	25.98	0.76	1.37	20.00
4	23.32	15.59	19.49	0.57	1.03	40.00

**Table 2:** Actual composition of MEA/(TiC)x (x = 0, 0.1, 0.2, 0.4).

Sample	Elemental Composition, wt.%					
	Co	Cr	Ni	Al	Ti	C
Alloy 1	43.50	22.65	27.28	1.08	1.90	3.59
Alloy 2	33.21	16.97	28.60	1.01	11.80	8.41
Alloy 3	28.04	15.85	22.40	0.80	20.99	11.56
Alloy 4	17.54	11.21	14.20	0.55	39.05	17.44

**Table 3:** Porosity of MEA/(TiC)x (x = 0, 0.1, 0.2, 0.4).

Value	Sample			
	Alloy 1	Alloy 2	Alloy 3	Alloy 4
Dry weight, g.	20.724	22.092	25.276	20.52
Floating weight, g.	2.740	3.121	3.736	3.675
Wet weight, g.	20.730	22.218	25.429	21.194
Porosity, %	0.33	0.56	0.71	3.84

**Table 4:** The carbide proportion of alloys with different TiC content.

Sample	Theoretical TiC Fraction, vol. %	Calculated Area Fraction of TiC, %	Maximum Size of TiC, μm.	Average Size of TiC, μm.
Alloy 2	13.83	14.79	11.53	2.52
Alloy 3	27.69	25.43	18.88	2.71
Alloy 4	48.28	44.59	28.26	3.01

**Table 5:** Average friction coefficients of MEA/(TiC)x (x = 0, 0.1, 0.2, 0.4).

Normal Force, N	Alloy 1	Alloy 2	Alloy 3	Alloy 4
2	0.5035	0.5654	0.5424	0.2251
5	0.5090	0.5804	0.5596	0.2137
10	0.4957	0.6203	0.6109	0.5730

**Table 6:** Wear MEA/(TiC)x (x = 0, 0.1, 0.2, 0.4) scar depth.

Normal Force, N	Depth, μm			
	Alloy 1	Alloy 2	Alloy 3	Alloy 4
2	17.498	4.683	4.046	0.378
5	21.925	20.679	10.729	0.395
10	39.544	39.053	19.811	1.299

**Table 7:** Wear MEA/(TiC)x (x = 0, 0.1, 0.2, 0.4) scar width.

Normal Force, N	Width, mm			
	Alloy 1	Alloy 2	Alloy 3	Alloy 4
2	0.687	0.419	0.315	0.110
5	0.740	0.679	0.668	0.146
10	1.010	0.991	0.854	0.474

## Data Availability

The data supporting this study’s findings are available from the corresponding author upon reasonable request. The data are not publicly available due to [The original contributions presented in the study are included in the article, further inquiries can be directed to the corresponding author/s].
